# Cardiopulmonary exercise testing and echocardiographic exam: an useful interaction

**DOI:** 10.1186/s12947-019-0180-0

**Published:** 2019-12-03

**Authors:** Ciro Santoro, Regina Sorrentino, Roberta Esposito, Maria Lembo, Valentina Capone, Francesco Rozza, Massimo Romano, Bruno Trimarco, Maurizio Galderisi

**Affiliations:** Department of Advanced Biomedical Sciences, Federico II University Hospital, Naples, Italy

**Keywords:** Cardiopulmonary exercise test, Echocardiography, Stress echo, Heart failure, Exercise prescription, Cardiomyopathies, Pulmonary hypertension, Coronary artery disease

## Abstract

Cardiopulmonary exercise test (CPET) is a functional assessment that helps to detect disorders affecting the system involved in oxygen transport and utilization through the analysis of the gas exchange during exercise. The clinical application of CPET is various, it including training prescription, evaluation of treatment efficacy and outcome prediction in a broad spectrum of conditions. Furthermore, in patients with shortness of breath it provides pivotal information to bring out an accurate differential diagnosis between physical deconditioning, cardiopulmonary disease and muscular diseases. Modern software allows the breath-by-breath analysis of the volume of oxygen intake (VO_2_), volume of carbon dioxide output (VCO_2_) and expired air (VE). Through this analysis, CPET provides a series of additional parameters (peak VO_2_, ventilatory threshold, VE/VCO_2_ slope, end-tidal carbon dioxide exhaled) that characterize different patterns, helping in diagnosis process. Limitations to the routine use of CPET are mainly represented from the lack of measurement standardization and limited data from randomized multicentric studies. The integration of CPET with exercise stress echocardiography has been recently introduced in the clinical practice by integrating the diagnostic power offered by both the tools. This combined approach has been demonstrated to be valuable for diagnosing several cardiac diseases, including heart failure with preserved or reduced ejection fraction, cardiomyopathies, pulmonary arterial hypertension, valvular heart disease and coronary artery disease. Future investigations are needed to further promote this intriguing combination in the clinical and research setting.

## Introduction

Cardiopulmonary exercise testing (CPET) allows the evaluation of gas exchange throughout exercise, providing a detailed description about the system involved in both O_2_ transport and its utilization during exercise. This information has a critical practical relevance in different clinical settings since CPET provides data on functional capacity, training prescription [1], treatment efficacy and outcome prediction in a broad spectrum of conditions [2–4]. Now days, this test has achieved relevant impact in clinical decision making [5], obtaining class I recommendation for evaluating exertion dyspnoea of uncertain cause and stratifying cardiac risk before heart transplant in heart failure [6]. Shortness of breath may represent the expression of different circumstances, ranging from physical deconditioning to cardiopulmonary or muscular diseases. When first line exams such as standard exercise testing, echocardiography or spirometry, have not identified a definite cause of this clinical symptom, CPET should be considered. Given its high negative predictive value [7], normal CPET response may exclude clinically significant heart diseases. This technique remains largely underused in the clinical setting, mainly in relation with the poor knowledge of its evidences and potentialities. Moreover, little is known about its interaction with echocardiography in diagnosing and managing heart failure patients.

Accordingly, the purpose of this review was to spread awareness about the distinct clinical impact of CPET and its interaction with the echocardiographic exam findings, a combination which can substantially improve the patient’s management in a variety of different conditions.

## Methodology of CPET

**Fig. 1:**
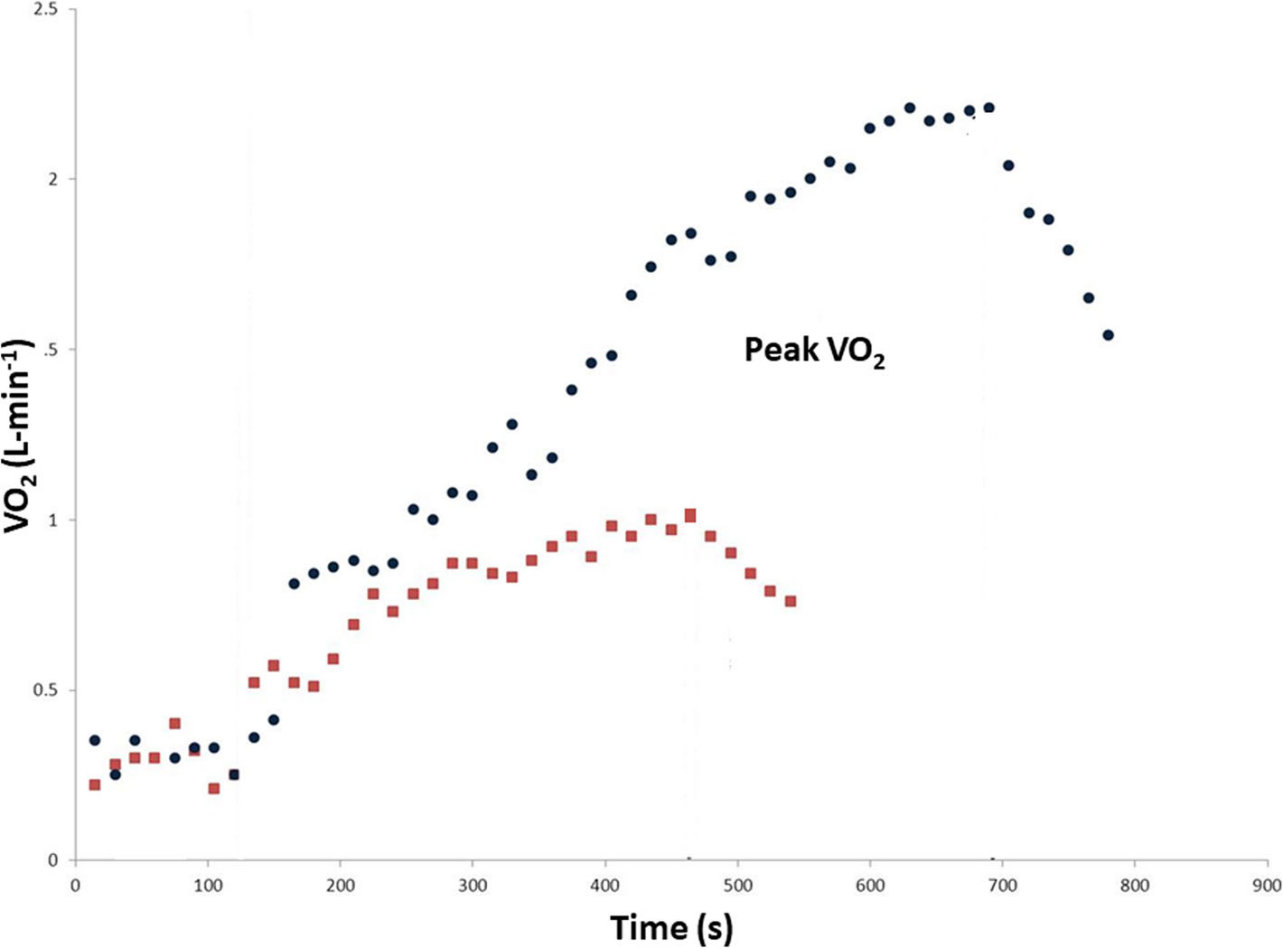
Oxygen uptake pattern during CPET ramp protocol. The blue dotted line represents a normal pattern. The red dotted line is representative of a patient with heart failure with a resulting reduced peak VO_2_

CPET can be performed on both cycle-ergometer or treadmill according to the individual laboratory availability. Data on ventilation and respiratory gas exchange can be collected by using a facemask or a mouthpiece. CPET is usually carried out using an incremental-work approach based on a ramp-like protocol. Ramp protocol consists in a gradual raise of work rate within each minute during the exercise [8], avoiding abrupt increases occurring in step-like protocol. By using this approach, a more linear and physiological response to the test is obtained, providing a more readable results. Accordingly, CPET allows to precisely determine at which level of effort the symptoms occur, and whether this happens before or after the anaerobic threshold. Frequently, a 10-watts per minute (W/min) ramp protocol with 1 W per 6 s work rate increment is used in the clinical setting (Fig. 1).

## CPET variables interpretation

Modern softwares allow the breath-by-breath analysis of the volume of oxygen intake (VO_2_), volume of carbon dioxide output (VCO_2_) and expired air (VE). Through this analysis, CPET provides a series of parameters that characterize different patterns, helping in diagnosis process. Table 1 reports common parameters resulting from CPET.

**Table 1:** Parameters of CPET and normal values

Variables	Meaning	Normal values
Peak VO2	Highest oxygen uptake (aerobic capacity)	> 85% of predicted Varies with age sex activity level, weight, use of betablockers
Ventilatory threshold (VT)	Represents the moment at which anaerobic metabolism increases (aerobic-anaerobic switch)	Between 40 to 60% of peak VO2
Ventilatory volume/carbon dioxide output (VE/VCO_2_) slope	Corresponds to ventilatory efficiency	Between 25 and 30
Peak respiratory exchange ratio (VCO_2_/VO_2_)	Reflects metabolism	< 0.8 at rest > 1.1 physiological maximal effort
Peak Heart rate	Chronotropic competence	Peak rate > 85% of the predicted
Heart rate recovery	Maximum HR minus HR at 1-min recovery	> 12 bpm
End-tidal PCO_2_	Identifies the perfusion state	> 33 mmHg at rest > 36 mmHg during exercise
O_2_ uptake efficiency slope	Additional logarithmic model of ventilatoryefficiency	< 1.4
Peak VE/Maximal voluntary ventilation (MVV)	Reflects the ventilatory reserve	15–20%

VO_2_ is a pivotal parameter that embodies insights on both cardiac and pulmonary function as an expression of the Fick’s principle according to which VO_2_ corresponds to cardiac output multiplied by the artero-venous gradient [C(a-v)O_2_]. During ramp-like exercise VO_2_ increases exponentially up to a steady state corresponding to peak exercise. Three abnormal patterns of VO_2_ curve can be observed during ramp test. The first is the upward shift of the overall curve due to higher request of O_2_ consumption as it happens in obese patients. The second is a relatively shallow slope secondary to reduced oxidative enzyme activity in skeletal muscle due to chronic heart failure or deconditioning. The third pattern, known as “*the hockey stick*” pattern, i.e. ΔVO_2_/Δwork rate (WR) flattening, is represented by a sharp and sudden interruption of the slope anticipating the expected peak intensity. The sudden interruption of oxygen uptake during the exercise is due to the exhaustion of the patient’s energy reserve, which is typical of myocardial ischemia, diastolic or systolic dysfunction, valve regurgitation or of conditions in which the exercise related heart rate increase is blunted by beta-blockers [9].

Peak VO_2_ corresponds to the peak values of oxygen consumption at maximal effort, expressed by litres of oxygen per minute or indexed as millilitres of oxygen per kilogram of body weight per minute. It describes the maximal amount of energy produced by aerobic metabolism. Peak VO_2_ can be reported also as a percentage of predicted peak VO_2_. Predicted pre-testpeak VO_2_changes according to age and sex have been established, they being lower in the elderlyand in female patients [10, 11].

Ventilatory threshold (VT) corresponds to the point at which muscle oxygen demand is higher than oxygen delivery, so that the metabolism switches from aerobic to anaerobic. This parameter is usually indirectly derived from VO_2_, VCO_2_ and VE data, but can even be directly obtained measuring blood lactate levels. In healthy subjects the ventilatory threshold usually occurs in between 40 and 60% of peak VO_2_ [12]. Values of ventilatory threshold are lower than those predicted in case of cardiopulmonary disease or deconditioning. When metabolism becomes mainly anaerobic, the lactic acid produced at this point is buffered by bicarbonate anions, thus increasing the level of carbon dioxide exhaled. As a result, the ratio between exhaled CO_2_ and the oxygen uptake (peak respiratory exchange ratio) increases. Therefore, values of peak respiratory exchange ratio above 1.1 during exercise identify a consistent anaerobic metabolism activation. Additionally, since high VCO_2_/VO_2_ ratio is an expression of the exercise burden, this parameter is also used to double-check if the effective patient’s motivation is enough elevated to accomplish the maximal effort (only in presence of an elevated VCO_2_/VO_2_, a stress test can be considered to be maximal). Exercise interruption at a peak respiratory exchange ratio lower than 1.0 can express limitation in muscle strain, possibly hiding hemodynamic or ventilatory impairment.

**Fig. 2:**
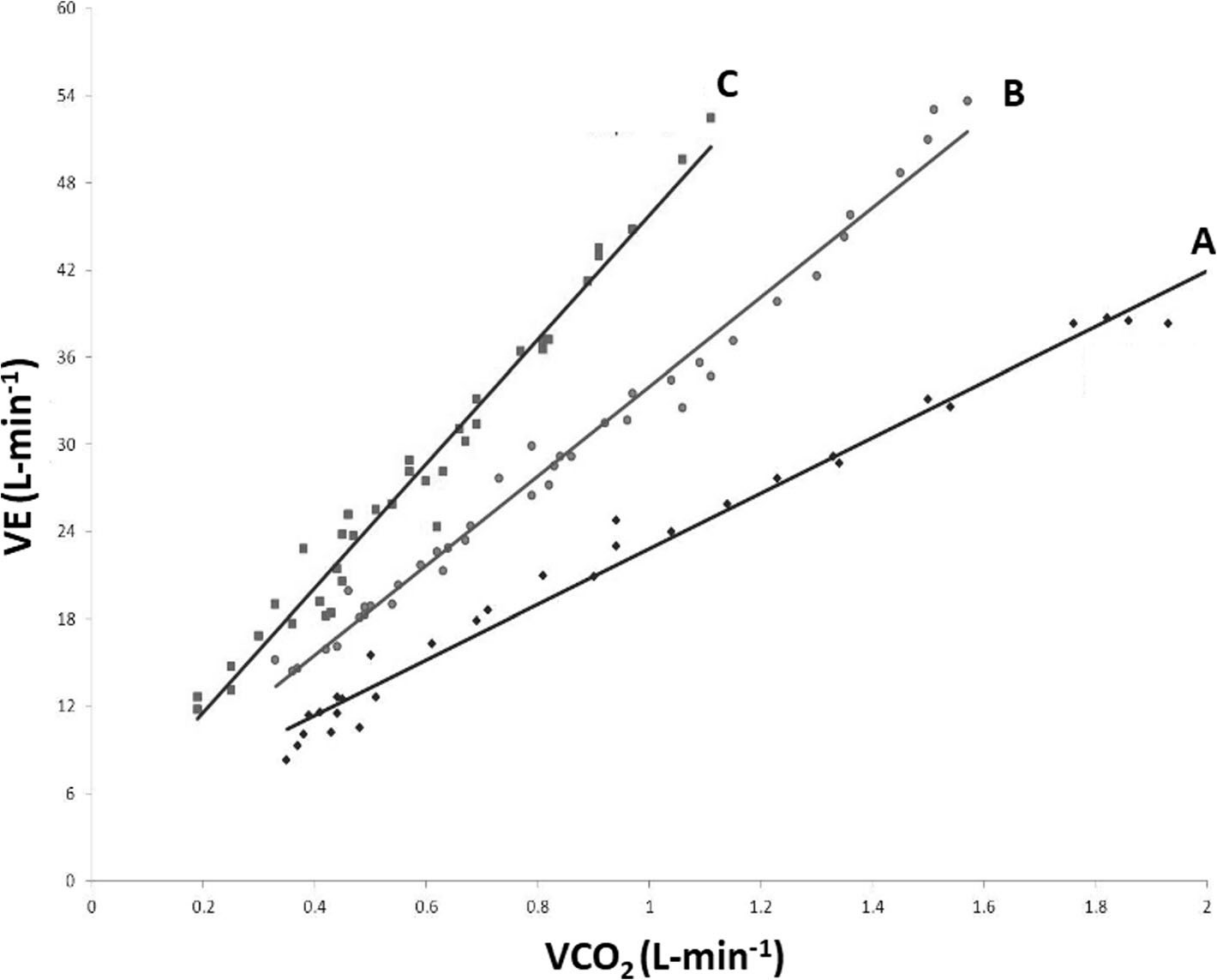
The VE/VCO_2_ slope during ramp incremental exercise in a normal subject (**a**) and in a patient with mild (**b**) and moderate (**c**) heart failure. A reduced ventilatory efficiency is present in heart failure expressed by a steeper VE/Vco_2_ slope when compared with that of a normal subject. VE = Ventilation; VCO_2_ = Volume of exhaled carbon dioxide; HF = Heart failure patient

VE/VCO_2_ slope represents the ventilatory efficiency, measuring the amount of exhaled air needed to expel one litre of carbon dioxide. Regularly, VE/VCO_2_ slope increases with age and is altered by ventilation perfusion mismatch following cardiopulmonary or metabolic disease. Worthy of note, among the different CPET parameters, VE/VCO_2_ appeared to be the only one capable of predicting prognosis in patients with diastolic heart failure [13] (Fig. 2).

The partial pressure of end-tidal carbon dioxide exhaled (end-tidal PCO_2_) identifies the perfusion state, or more precisely is a parameter of ventilation/perfusion mismatch (V/Q mismatch). It inversely correlates with cardiac output [14], being markedly reduced in conditions of circulatory impairment, as it occurs in chronic heart failure because of a higher V/Q mismatch. However, end-tidal PCO_2_can be reduced also in respiratory dysfunction in which alveolar dead space is increased, such as pulmonary emphysema or parenchymal lung diseases, independently of the state of cardiac function [15].

Other quantitative parameters can be analysed during CPET, such as oscillatory ventilation expressing ventilation fluctuation during exercise. Oscillatory ventilation can be due either to ventilatory or hemodynamic instability [16]. Oscillatory ventilation pattern is recognized when it involves more than 60% of the exercise duration with 15% of variation compared to ventilation values at rest [6]. The oxygen uptake efficiency slope (OUES) is derived from the relationship between VO_2_ and the log transformation of VE and expresses the ventilatory requirement for a given O_2_ [6].

## Clinical applications

### Exercise prescription

CPET is considered an accurate method to assess aerobic performance for both healthy individuals and patients with cardiovascular and/or respiratory diseases, consistently driving the exercise prescription [17]. Pivotal data in exercise prescription are heart rate (HR) and VT. The exercise performed below VT is considered the sub-maximal level tolerated by an individual patient for a sustained amount of time. Moreover, HR values at different points through the exercise are reported (i.e. HR at rest, HR at VT) in order to refine aerobic exercise prescriptions.

### CPET in heart failure

Functional assessment measured by CPET gives pivotal information about maximal aerobic capacity, therapy management and exercise prescription in patients with chronic heart failure. In the majority of these patient, CPET shows reduced VO_2_, VT < 40% of the predicted VO_2_ curve, peak VO_2_ < 85%, increased VE/VCO_2_, but normal O_2_ saturation [18]. Of interest, peak VO_2_ < 14 mL/kg/min carries a poor prognosis, being considered as indication for heart transplant [19]. Combined all together, these parameters, along with wide oscillations in ventilation during exercise and low HR recovery during the first minute after peak stress, reflect the ventilatory and metabolic inefficiency and are of relevant impact on prognosis in heart failure patients [20]. A comprehensive analysis of these parameters can help in accurately predicting the mortality rate in these patients [21]. In a metanalysis of studies on patients with heart failure undergoing CPET, peak VO_2_, VE/VCO_2_ slope, OUES and periodic ventilation appeared to have a strong prognostic impact, predicting adverse cardiovascular events with odds ratios of 4.10 (CI: 3.16–5.33), 5.40 (CI: 4.17–6.99), 8.08 (CI: 4.19–15.58) and 5.48 (CI: 3.82–7.86), respectively [22]. Myers et al. produced a stratification score that integrates most of the above-mentioned parameters (Table 2). The score ranges from 0 to 20, with the first group (0–5) used as a reference. Patients with a score >  15 had a 3 years mortality of 12.2% [23]. Noteworthy, VT can be undetermined in patients with considerably reduced exercise tolerance, thus unidentifiable VT is also considered a negative prognostic factor in patients with end-stage heart failure [24]. Accordingly, CPET has class I recommendation and level A in patients with HFrEF being considered for heart transplantation or mechanical device implantation [6]. In heart failure with preserved ejection fraction (HFpEF), not only peak VO_2_, but also the percent-predicted peak VO_2_ appear not be able to predict adverse events, probably, because current algorithms work poorly in this clinical setting. However, VE/VCO_2_ has shown the capability of predicting adverse events [25, 26] In particular, a VE/VCO_2_ slope > 33.3 showed a sensitivity of 97% and a specificity of 40% in predicting mortality and cardiac-related hospitalization in patients left ventricular ejection fraction (LVEF) > 50% [13].

**Table 2:** Cardiopulmonary exercise test score (modified from Ref # 23)

Variable	Value	Points
VE/VCO_2_slope	≥34	7
HR recovery	≤6	5^a^
O2 uptake efficiency slope	≤1.4	2
Peak VO_2_	< 14 mL/Kg/min	2

Score > 15 points: annual mortality rate 12.2%

^a^2 point if undergoing beta-blocker therapy

### CPET in differential diagnosis of dyspnoea

In the cases of unexplained dyspnoea, 4 different categories can be identified by combining CPET variables: cardiac, pulmonary, mixed and non-cardiopulmonary [27, 28]. Reduction in peak VO_2_ is seen in both respiratory, cardiac and metabolic disease. Mainly, patients with respiratory diseases show a significant drop (i.e.,> 4% on peak exertion) in O_2_ saturation and low breathing reserve (i.e.,< 20%) [29]. On the other hand, patients with exertion dyspnoea induced by cardiac diseases show reduced peak VO_2_, early VT, high VE/CO_2_ slope, reduced OUES [29]. Of note, OUES has gained a recognized prognostic value in patients undergoing submaximal exercise [6]. In both primary or thromboembolic pulmonary arterial hypertension (PAH), low peak VO_2_ and high VE/Vco_2_ ratio during exercise have demonstrated to be useful in establishing the severity of functional impairment [30]. Consistently, CPET can be of helpful for the physicians who must face patients complaining dyspnoea both in terms of differential diagnosis and symptoms classification. Table 3 summarizes abnormal CPET patterns in patients with dyspnoea.

**Table 3:** CPET variables in different causes of dyspnea

Condition	Variables
Cardiovascular	Peak VO_2_ < 80% of the predicted
	Low ventilatory threshold (VT)
	Chronotropic incompetence
	Heart rate recovery ≤12 BPM after the first minute
Pulmonary	Peak VO_2_ < 80% of the predicted
	Low ventilatory threshold (VT)
	Peak respiratory rate > 50/min
	Ventilatory reserve (peak VE/MVV) < 15%
	Oxygen desaturation
Deconditioning	Low-normal peak VO_2_
	Low ventilatory threshold (VT)
	Absence of any other abnormal response
Obesity	Absolute VO_2_ greater than predicted
	Indexed peak VO_2_ lower than predicted
	Increased VO_2_/work slope
Muscle disease	Submaximal cardiac and respiratory response
	Low ventilatory threshold (VT)
	Elevate lactate at submaximal work

### CPET in congenital heart disease

CPET provides an integrated evaluation of cardiac, pulmonary, and metabolic function and may be used to identify the source of exercise limitation in congenital heart disease. Because CPET measurements have also been associated with outcome in adults with congenital heart disease, CPET is now considered as an important prognostic indicator and also useful for surgical stratification in this population [31].

## Integration of CPET and echocardiography

### Heart failure

Exercise stress echocardiography (ESE) and CPET can be considered an intriguing combination, possibly providing fundamental information on differential diagnosis and therapeutic management in patients suffering for exertion dyspnoea in different clinical settings, mainly in patients complaining heart failure symptoms and valve heart disease. The combination CPET-ESE can non-invasively evaluate multiple aspects of the cardiovascular system, offering a more personalised O_2_ pathway analysis, which is otherwise obtainable only with invasive hemodynamic monitoring [32]. In this context, the CPET-ESE approach is particularly valuable in identifying non-cardiopulmonary causes of dyspnoea, which are mainly related to an impaired oxygen extraction (AVO_2_diff) [5]. Different authors have demonstrated that the effort intolerance observed in HFpEF and heart failure with mid range LVEF could be related to an impaired AVO_2_diff (peripheral component of Fick equation) and near-normal cardiac output [33–35].

**Fig. 3:**
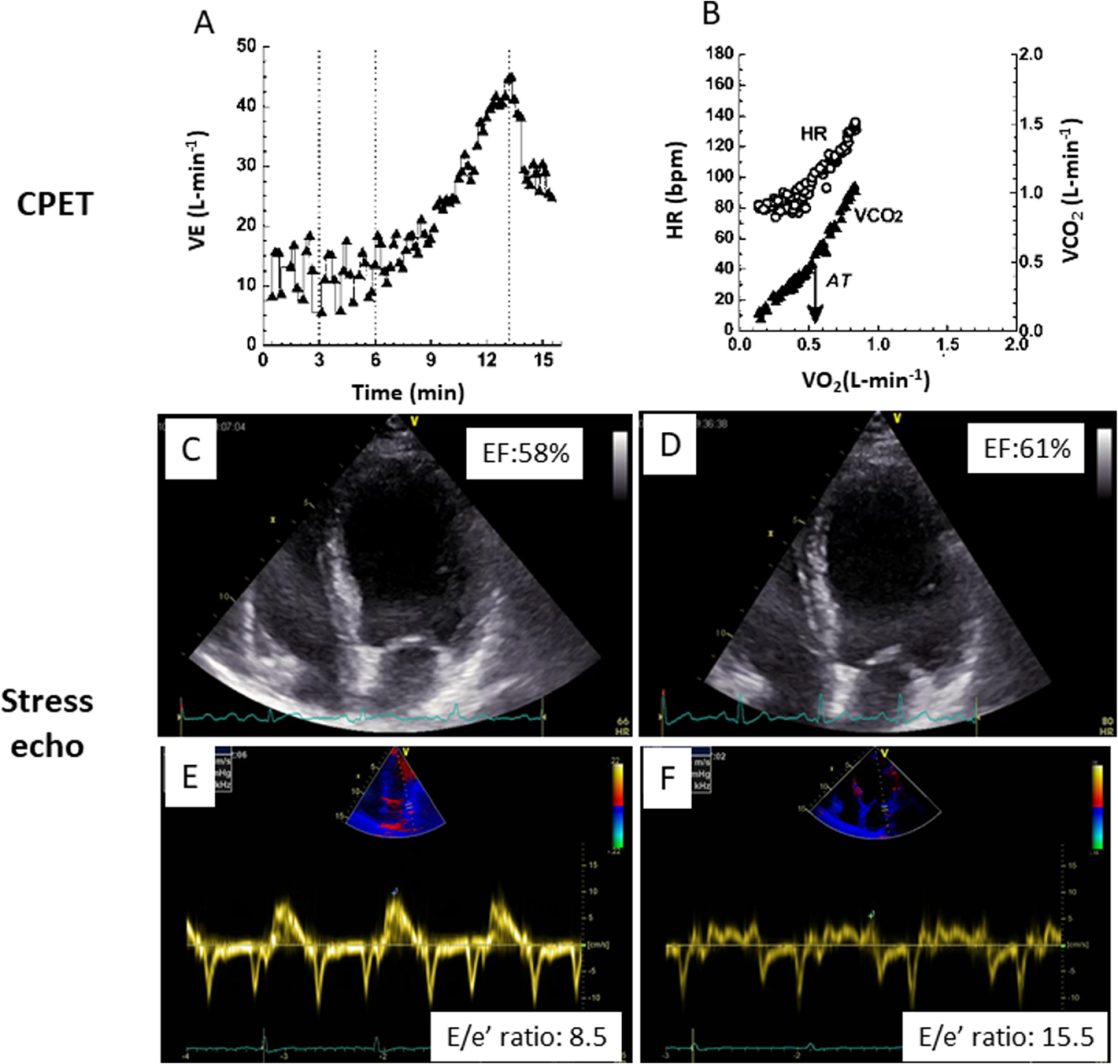
Illustrative clinical case of combined CPET and stress echo approach in a patient affected by HFpEF. CPET analysis shows clear oscillatory patterns of minute ventilation (VE) (**a**) and reduced VE/VCO_2_ ratio (**b**). Echocardiographic exam at rest shows a preserved ejection fraction (**c**) and an E/e’ ratio in the normal range (**e**). At peak exercise the ejection fraction is normal (**d**) but E/e’ appears to be pathologically increased (**f**)

In some patients, complaining exertion dyspnoea, in particular if hypertensive, the early stages of HFpEF cannot be always detectable by the sole echocardiographic exam at rest since the simple quantification of LVEF often fails to predict functional capacity. Under these circumstances, the combination of speckle tracking echocardiography and CPET may provide additional information. Global longitudinal strain (GLS) is reduced in parallel with a reduced peak VO_2_ response and was superior to LVEF in identifying patients with impaired peak VO_2_ [36]. A comprehensive non-invasive evaluation of LV diastolic function – performed according to standardized ASE/EACVI recommendations [37] - has also a proved a diagnostic impact in predicting functional capacity in patients with HFpEF [34]. Since patients with normal LV filling pressures or even normal LV diastolic function at rest may reveal elevated LV filling pressures during effort [37–41], diastolic stress testing is indicated when echo exam at rest does not explain the symptoms of heart failure or dyspnoea, especially with exertion [37]. An E/e’ ratio >  15 during exercise can be considered as an accurate marker of HFpEF in presence of cardiac symptoms [42–45]. Accordingly, the combination of CPET results, in particular VE/CO_2_ slope, and E/e’ ratio at peak stress may be highly demonstrative of HFpEF (Fig. 3) [46]. This is confirmed also in patients with ischemic heart failure in which E/e’ ratio at peak stress was the most useful parameter for identifying severe exercise intolerance, as indicated by peak oxygen uptake < 14 mL/kg/min (AUC of E/e’ ratio ≥ 18 = 0.92, sensitivity = 85.2%, specificity = 95.6%) [47]. Worthy of note, the integrated CPET-ESE approach proved to increase patient risk stratification also in HFrEF, thanks to possibility of directly studying both LV and right ventricular (RV) contractility [35, 48].

### Valvular heart disease

Given the complicated relationships existing between hemodynamic changes from resting condition to peak exercise in patients with valvular disease, new protocols combining ESE and CPET may give detailed information to better face the challenge in developing optimal individualized therapy [49]. ESE associated with CPET can provide crucial information on exercise intolerance in asymptomatic patients with hemodynamically significant mitral regurgitation (MR). Reduced peak VO_2_ has an important prognostic value in patients with significant MR, although the mechanisms underlying this association are not well established. In this subset of patients, ESE can provide information about the hemodynamic response to effort by measuring mean pulmonary arterial pressure (PAPm), systolic pulmonary arterial pressure (PAPs), RV systolic function and cardiac output (CO). Recently, reduced values in pulmonary vascular reserve, measured by PAPm/CO slope, and in RV contractile reserve, expressed by tricuspid annulus plane systolic excursion (TAPSE)/PAPs changes between rest and peak effort, were found to predict a low peak VO_2_ response during effort. Accordingly, this association may explain the etiology of impaired exercise tolerance in patients affected by asymptomatic but significant MR. The combination of low pulmonary vascular reserve, impaired RV contractile reserve and low peak VO_2_ may also guide the optimal timing for mitral valve surgery [50]. Frequently, patients with mitral stenosis (MS) show reduced exercise tolerance that, in some cases, is out of proportion compared to the hemodynamic at rest [49]. It is conceivable that several factors could contribute to alter exercise response in MR. Indeed, a low peak exercise HR (chronotropic incompetence) and the absence of a significant rise in stroke volume (impaired contractile reserve), combined with a reduced respiratory reserve (restrictive lung function) have a critical impact on the exercise response in MS. Accordingly, by combining CPET with echocardiography it is possible to identify the different determinants of reduction of both exercise capacity and peak VO_2_, thus improving patient selection for targeted treatment. Of note, Laufer-Perl et al. demonstrated that in patients with moderate-to-severe MS, restrictive lung function, chronotropic incompetence and limited contractile reserve had a greater impact on symptoms compared to MS severity itself, as expressed by the transvalvular gradient and the mitral valve area [51].

### Primary cardiomyopathies

Another possible combination of CPET and echocardiography involves cardiomyopathies and, in particular, the differential diagnosis with the athlete’s heart. Echocardiography is largely used for diagnosis of hypertrophic cardiomyopathy (HCM), it allowing to characterize a disproportionate increase of LV wall thickness and a reduction of LV end-diastolic diameter [51]. However, maximal wall thickness ranging between 13 and 15 represents a grey zone which can occur in 4% of males and more frequently in black athletes [52]. In addition, diagnostic accuracy of echocardiography is limited by the lack of clear cut-off points stratified by ethnicity, gender and sport types. CPET can help the echo approach to appropriately diagnosing HCM in athletes [52]. VO_2_max resulted to be substantially reduced in athletes with HCM than in healthy athletes; in particular, a pVO2 > 50 ml/kg/min or >  20% above the predicted maximum VO_2_ differentiated athlete’s heart from HCM [53]. These results could open unexplored horizons in order to refine echocardiographic diagnosis of HCM in athletes.

### Pulmonary arterial hypertension

In chronic thromboembolic PAH, a fast and accurate diagnosis is pivotal for successful treatment. Clinical symptoms/signs may be nonspecific and risk factors not always detectable. Echocardiography is the recommended first-line diagnostic tool and guidelines recommend non invasively estimation of PAPs (by peak velocity of tricuspid regurgitation and atrio-ventricular pressure gradient) and detection of indirect signs of PAH (RV and right atrial dilation, RV systolic dysfunction corresponding to a reduced TAPSE and standard Doppler derived abnormalities of RV outflow tract) [54, 55]. CPET may be complementary and help to identify patients with milder abnormalities and chronic thromboembolic disease. Patients with impaired ventilation due to pulmonary arterial obstruction show elevated alveolar-capillary gradients of O_2_ and CO_2_ [56]. In a retrospective report, CPET was able to identify chronic thromboembolic PAH, despite normal echo exam [57]. It is also worthy of note that In patients symptomatic for dyspnea, the occurrence of ΔVO_2_/Δwork rate flattening, ie. the “hockey stick” pattern, demonstrated to reflect a significantly impaired functional phenotype whose major cardiac determinants are the excessive PAPs increase and the reduced TAPSE) [58].

### Coronary artery disease

In the setting of coronary artery disease, the combination of ESE and CPET performed in 110 patients, allowed to discriminate between coronary circulatory disease and de-conditioning (i.e., a decrease in the responsiveness of heart muscle occurring after long periods of weightlessness and corresponding to a blood volume reduction and blood pooling in the legs upon return to normal conditions) [59]. In fact, multiple gas exchange parameters obtained by CPET were associated, despite with low sensitivity, with abnormal echo-Doppler derived stroke volume response to stress, and VE/VCO_2_ slope to peak VO_2_ ratio was the best discriminator (≥2.7: AUC 0.79, *p* < 0.0001). These findings demonstrate that in patients with borderline results, a combined stress-echo with CPET, measuring stroke volume and A-VO_2_ difference throughout effort may be helpful for diagnosing significant coronary artery disease. Furthermore, stress echo derived wall motion abnormalities of isolated coronary lesions other than anterior descending artery, may require particular effort due to poor endocardial visualization, particularly when dealing with significant lesion of the right coronary artery. Blunted physiological VO_2_ increase and plateau in HR response during CPET has demonstrated to be indicative of myocardial ischemia of right coronary artery, anticipating ECG abnormalities [60]. Hence, we can speculate that combined analysis of CPET pattern and wall motion abnormalities during ESE may improve the accuracy level in diagnosing right coronary artery stenosis.

Table 4 reports the main echo-derived systolic and diastolic measurement which can be combined with CPET parameters.

**Table 4:** ESE parameters and normal values

Variables	Meaning	Normal values
Δ LVEF	Contractile reserve	> 5%
ΔGLS	Contractile reserve	> 2%
ΔSV	Contractile reserve	> 20%
Peak E/e’	Elevated LV filling pressure during stress	> 15
Peak PAPs	Maximal pulmonary systolic pressure during stress	> 60 mmHg
ΔEROA	Changes in mitral regurgitation severity during time	< 10 mm^3^
ΔTransmitral MPG	Changes in transmitral pressure gradient during stress	< 15 mmHg
ΔTransaortic MPG	Changes in transaortic pressure gradient during stress	< 20 mmHg
LVOT Maximal Peak Gradient	In case of LVOT obstruction it reflects pathological	< 50 mmHg – low prognostic impact

*LVEF* Left ventricular ejection fraction, *GLS* Global longitudinal strain, *SV* Stroke volume, *EROA* Effective regurgitant orifice area, *MPG* Mean pressure gradient, *LVOT* Left ventricular output tract

## Conclusions

CPET is being increasingly applied together with echocardiography, in particular ESE, in order to combine functional and structural data. Its use may add crucial information to the echo exam, in particular during stress. The additional diagnostic value of this combined assessment has been demonstrated in multiple clinical settings, including heart failure, valvular heart disease, hypertrophic cardiomyopathy, chronic thromboembolic derived PAH and coronary artery disease. On the grounds of recognized evidences [23, 61], it is conceivable that CPET data combined with clinical, laboratory and echocardiographic measurements could very efficiently stratify prognosis in patients with cardiac diseases.

## Data Availability

Not applicable.

## Abbreviation

C(a-v)O_2_: Arterial-venous gradient
CPET: Cardiopulmonary exercise test
ESE: Exercise stress echocardiography
HCM: Hypertrophic cardiomyopathy
HFpEF: Heart failure with preserved ejection fraction
HR: Heart rate
LV: Left ventricular
OUES: Oxygen uptake efficiency slope
PAH: Pulmonary arterial hypertension
PAPm: Pulmonary arterial mean pressure
PAPs: Pulmonary arterial systolic pressure
TAPSE: Tricuspid annulus plane systolic excursion
VCO_2_: Volume of carbon dioxide output
VE: Volume of expired air
VO_2_: Volume of oxygen intake
VT: Ventilatory threshold

## References

[1] Vesterinen V, Nummela A, Heikura I, Laine T, Hynynen E, Botella J, Häkkinen K (2016). Individual endurance training prescription with heart rate variability. Med Sci Sports Exerc.

[2] Corrà U, Piepoli MF, Adamopoulos S, Agostoni P, Coats AJ, Conraads V (2014). Cardiopulmonary exercise testing in systolic heart failure in 2014: the evolving prognostic role: a position paper from the committee on exercise physiology and training of the heart failure association of the ESC. Eur J Heart Fail.

[3] Guazzi M, Arena R, Halle M, Piepoli MF, Myers J, Lavie CJ (2016). 2016 focused update: clinical recommendations for cardiopulmonary exercise testing data assessment in specific patient populations. Circulation.

[4] Myers J (2005). Applications of cardiopulmonary exercise testing in the management of cardiovascular and pulmonary disease. Int J Sports Med.

[5] Guazzi M, Bandera F, Ozemek C, Systrom D, Arena R (2017). Cardiopulmonary exercise testing: what is the value ?. J Am Coll Cardiol.

[6] Gibbons RJ, Balady GJ, Bricker JT, Chaitman BR, Fletcher GF, Froelicher VF (2002). American College of Cardiology/American Heart Association Task Force on Practice Guidelines (Committee to Update the 1997 exercise testing guidelines). ACC/AHA 2002 guideline update for exercise testing: summary article: a report of the American College of Cardiology/American Heart Association task force on practice guidelines (committee to update the 1997 exercise testing guidelines). Circulation.

[7] Nusair S (2017). Interpreting the incremental cardiopulmonary exercise test. Am J Cardiol.

[8] Myers J, Bellin D (2000). Ramp exercise protocols for clinical and cardiopulmonary exercise testing. Sports Med.

[9] Adachi H (2017). Cardiopulmonary Exercise Test. Int Heart J.

[10] Betik AC, Hepple RT (2008). Determinants of VO_2_max decline with aging: an integrated perspective. Appl Physiol Nutr Metab.

[11] Astrand I (1960). Aerobic work capacity in men and women with special reference to age. Acta Physiol Scand.

[12] Wasserman K, Hansen JE, Sue DY, Stringer WW, Whipp BJ, Wasserman K, Hansen JE, Sue DY, Stringer WW, Sietsema KE, Sun X-G, Whipp BJ (2012). Normal values. Principles of exercise testing and interpretation. Including pathophysiology and clinical applications.

[13] Guazzi M, Myers J, Arena R (2005). Cardiopulmonary exercise testing in the clinical and prognostic assessment of diastolic heart failure. J Am Coll Cardiol.

[14] Jin X, Weil MH, Tang W, Povoas H, Pernat A, Xie J, Bisera J (2000). End-tidal carbon dioxide as a noninvasive indicator of cardiac index during circulatory shock. Crit Care Med.

[15] Matsumoto A, Itoh H, Eto Y, Kobayashi T, Kato M, Omata M (2000). End-tidal CO_2_ pressure decreases during exercise in cardiac patients: association with severity of heart failure and cardiac output reserve. J Am Coll Cardiol.

[16] Khoo MC, Kronauer RE, Strohl KP, Slutsky AS (1982). Factors inducing periodic breathing in humans: a general model. J Appl Physiol.

[17] Araújo CG, Herdy AH, Stein R (2013). Maximum oxygen consumption measurement: valuable biological marker in health and in sickness. Arq Bras Cardiol.

[18] Herdy AH, Uhnlerdorf D (2011). Reference values for cardiopulmonary exercise testing for sedentary and active men and women. Arq Bras Cardiol.

[19] Mancini DM, Eisen H, Kussmaul W, Mull R, Edmunds LH, Wilson JR (1991). Value of peak exercise oxygen consumption for optimal timing of cardiac transplantation in ambulatory patients with heart failure. Circulation.

[20] Yancy CW, Jessup M, Bozkurt B, Butler J, Casey DE, Drazner MH (2013). American College of Cardiology Foundation; American Heart Association Task Force on Practice Guidelines. ACCF/AHA guideline for the management of heart failure: a report of the American College of Cardiology Foundation/American Heart Association task force on practice guidelines. J Am Coll Cardiol.

[21] Levy WC, Arena R, Wagoner LE, Dardas T, Abraham WT (2012). Prognostic impact of the addition of ventilatory efficiency to the Seattle heart failure model in patients with heart failure. J Card Fail.

[22] Cahalin LP, Chase P, Arena R, Myers J, Bensimhon D, Peberdy MA, Ashley E (2013). A meta-analysis of the prognostic significance of cardiopulmonary exercise testing in patients with heart failure. Heart Fail Rev.

[23] Myers J, Oliveira R, Dewey F, Arena R, Guazzi M (2013). Chase pet al. Validation of a cardiopulmonary exercise test score in heart failure. Circ Heart Fail.

[24] Agostoni P, Corrà U, Cattadori G, Veglia F, Battaia E, La Gioia R (2013). Prognostic value of indeterminable anaerobic threshold in heart failure. Circ Heart Fail.

[25] Malhotra Rajeev, Bakken Kristian, D’Elia Emilia, Lewis Gregory D. (2016). Cardiopulmonary Exercise Testing in Heart Failure. JACC: Heart Failure.

[26] Guazzi M (2014). Pulmonary hypertension in heart failure with preserved ejection fraction: pathophysiology and clinical perspectives. Circ Heart Fail.

[27] Wahls SA (2012). Causes and evaluation of chronic dyspnea. Am Fam Physician.

[28] Messner-Pellence P, Ximenes C, Brasileiro CF, Mercier J, Grolleau R, Prefaut CG (1994). Cardiopulmonary exercise testing: determinants of dyspnea due to cardiac or pulmonary limitation. Chest.

[29] Beaver WL, Wasserman K, Whipp BJ (1973). On-line computer analysis and breath-by-breath graphical display of exercise function tests. J Appl Physiol.

[30] Weatherald J, Farina S, Bruno N, Laveneziana P (2017). Cardiopulmonary exercise testing in pulmonary hypertension. Ann Am Thorac Soc.

[31] Khan AM, Paridon SM, Kim YY (2014). Cardiopulmonary exercise testing in adults with congenital heart disease. Expert Rev Cardiovasc Ther.

[32] Houstis NE, Eisman AS, Pappagianopoulos PP, Wooster L, Bailey CS, Wagner PD (2018). Exercise intolerance in heart failure with preserved ejection fraction: diagnosing and ranking its causes personalized O_2_ pathway analysis. Circulation.

[33] Dhakal BP, Malhotra R, Murphy RM, Pappagianopoulos PP, Baggish AL, Weiner RB (2015). Mechanisms of exercise intolerance in heart failure with preserved ejection fraction: the role of abnormal oxygen extraction. Circ Heart Fail.

[34] Shimiaie J, Sherez J, Aviram G, Megidish R, Viskin S, Halkin A (2015). Determinants of effort intolerance in patients with heart failure: combined echocardiography and cardiopulmonary stress protocol. JACC Heart Fail.

[35] Pugliese NR, Fabiani I, Santini C, Rovai I, Pedrinelli R, Natali A (2019). Value of combined cardiopulmonary and echocardiography stress test to characterize the hemodynamic and metabolic responses of patients with heart failure and mid-range ejection fraction. Eur Heart J Cardiovasc Imaging.

[36] Hasselberg NE, Haugaa KH, Sarvari SI, Gullestad L, Andreassen AK, Smiseth OA (2015). Left ventricular global longitudinal strain is associated with exercise capacity in failing hearts with preserved and reduced ejection fraction. Eur Heart J Cardiovasc Imaging.

[37] Nagueh SF, Smiseth OA, Appleton CP, Byrd BF, Dokainish H, Edvardsen T (2016). Recommendations for the evaluation of left ventricular diastolic function by echocardiography: an update from the American Society of Echocardiography and the European Association of Cardiovascular Imaging. Eur Heart J Cardiovasc Imaging.

[38] Oh KJ, Park SJ, Nagueh FS (2011). Established and novel clinical applications of the diastolic function assessment by echocardiography. Circ Cardiovasc Imaging.

[39] Burgess IM, Jenkins C, Sharman EJ, Marwick TH (2006). Diastolic stress echocardiography: hemodynamic validation and clinical significance of estimation of ventricular filling pressure with the exercise. J Am Coll Cardiol.

[40] Ha JW, Oh JK, Pellikka PA, Ommen SR, Stussy VL, Bailey KR (2005). Diastolic stress echocardiography: a novel noninvasive diagnostic test for diastolic dysfunction using supine bicycle exercise Doppler echocardiography. J Am Soc Echocardiogr.

[41] Agricola E, Oppizzi M, Pisani M, Margonato A (2004). Stress echocardiography in heart failure. Cardiovasc Ultrasound.

[42] Schiano-Lomoriello V, Santoro C, de Simone G, Trimarco B, Galderisi M (2015). Diastolic bicycle stress echocardiography: normal reference values in a middle age population. Int J Cardiol.

[43] Paulus WJ, Tschöpe C, Sanderson JE, Rusconi C, Flachskampf FA, Rademakers FE (2007). How to diagnose diastolic heart failure: a consensus statement on the diagnosis of heart failure with normal left ventricular ejection fraction by the heart failure and echocardiography associations of the European Society of Cardiology. Eur Heart J.

[44] Nedeljkovic I, Banovic M, Stepanovic J, Giga V, Djordjevic-Dikic A, Trifunovic D (2016). Combined exercise stress echocardiography and cardiopulmonary exercise test for identification of masked heart failure with preserved ejection fraction in patients with hypertension. Eur J Prev Cardiol.

[45] Arques S, Roux E, Luccioni R (2007). Current clinical applications of spectral tissue Doppler echocardiography (E/E' ratio) as a noninvasive surrogate for left ventricular diastolic pressures in the diagnosis of heart failure with preserved left ventricular systolic function. Cardiovasc Ultrasound.

[46] van Riel AC, Opotowsky AR, Santos M, Rivero JM, Dhimitri A, Mulder BJ (2017). Accuracy of echocardiography to estimate pulmonary artery pressures with exercise: a simultaneous invasive-noninvasive comparison. Circ Cardiovasc Imaging.

[47] Podolec P, Rubís P, Tomkiewicz-Pajak L, Kopeć G, Tracz W (2008). Usefulness of the evaluation of left ventricular diastolic function changes during stress echocardiography in predicting exercise capacity in patients with ischemic heart failure. J Am Soc Echocardiogr.

[48] Guazzi M, Villani S, Generati G, Ferraro OE, Pellegrino M, Alfonzetti E (2016). During exercise challenge in heart failure: pathophysiology and clinical phenotypes. JACC Heart Fail.

[49] Nishimura RA, Otto CM, Bonow RO, Carabello BA, Erwin JP, Fleisher LA (2017). 2017 AHA/ACC focused update of the 2014 AHA/ACC guideline for the Management of Patients with Valvular Heart Disease: a report of the American College of Cardiology/American Heart Association task force on clinical practice guidelines. J Am Coll Cardiol.

[50] Utsunomiya H, Hidaka T, Susawa H, Izumi K, Harada Y, Kinoshita M (2018). Exercise-stress echocardiography and effort intolerance in asymptomatic/minimally symptomatic patients with degenerative mitral regurgitation combined invasive-non invasive hemodynamic monitoring. Circ Cardiovasc Imaging.

[51] Laufer-Perl M, Gura Y, Shimiaie J, Sherez J, Pressman GS, Aviram G (2017). Mechanisms of effort intolerance in patients with rheumatic mitral stenosis: combined echocardiography and cardiopulmonary stress protocol. JACC Cardiovasc Imaging.

[52] Galderisi M, Cardim N, D'Andrea A, Bruder O, Cosyns B, Davin L (2015). The multi-modality cardiac imaging approach to the Athlete's heart: an expert consensus of the European Association of Cardiovascular Imaging. Eur Heart J Cardiovasc Imaging.

[53] Sharma S, Elliott PM, Whyte G, Mahon N, Virdee MS, Mist B (2000). Utility of metabolic exercise testing in distinguishing hypertrophic cardiomyopathy from physiologic left ventricular hypertrophy in athletes. J Am Coll Cardiol.

[54] Galiè N, Humbert M, Vachiery JL, Gibbs S, Lang I, Torbicki A (2016). 2015 ESC/ERS guidelines for the diagnosis and treatment of pulmonary hypertension. The joint task force for the diagnosis and treatment of pulmonary hypertension of the European Society of Cardiology (ESC) and the European Respiratory Society (ERS). Eur Heart J.

[55] Scheidl SJ, Englisch C, Kovacs G, Reichenberger F, Schulz R, Breithecker A (2012). Diagnosis of CTEPH versus IPAH using capillary to end-tidal carbon dioxide gradients. Eur Respir J.

[56] Serra W, Chetta A, Santilli D, Mozzani F, Dall'Aglio PP, Olivieri D (2010). Echocardiography may help detect pulmonary vasculopathy in the early stages of pulmonary artery hypertension associated with systemic sclerosis. Cardiovasc Ultrasound.

[57] Held M, Grün M, Holl R, Hübner G, Kaiser R, Karl S (2014). Cardiopulmonary exercise testing to detect chronic thromboembolic pulmonary hypertension in patients with normal echocardiography. Respiration.

[58] Bandera F, Generati G, Pellegrino M, Donghi V, Alfonzetti E, Gaeta M (2014). Role of right ventricle and dynamic pulmonary hypertension on determining DVO_2_/DVO_2_ work rate flattening: insights from cardiopulmonary exercise test combined with exercise echocardiography. Circ Heart Fail.

[59] Rozenbaum Z, Khouri S, Aviram G, Gura Y, Sherez J, Man A (2017). Discriminating circulatory problems from deconditioning: echocardiographic and cardiopulmonary exercise test analysis. Chest.

[60] Contini M, Andreini D, Agostoni P (2006). Cardiopulmonary exercise test evidence of isolated right coronary artery disease. Int J Cardiol.

[61] Agostoni P, Corrà U, Cattadori G, Veglia F, La Gioia R, Scardovi AB (2013). Metabolic exercise test data combined with cardiac and kidney indexes, the MECKI score: a multiparametric approach to heart failure prognosis. Int J Cardiol.

